# Design of Porous Aromatic Frameworks for Adsorptive Desulfurization: Synergistic Modulation via π-π Interactions and Mesopores

**DOI:** 10.3390/nano15231815

**Published:** 2025-11-30

**Authors:** Tiantian Li, Xiaowen Li, Hao Wu, Guangxia Shi, Yizhi Zeng, Dong Xu, Dingming Xue

**Affiliations:** 1 School of Chemical Engineering and Technology, Xuzhou College of Industrial Technology, Xuzhou 221140, China; 2 Xi’an Research Institute of High Technology, Xi’an 710025, China; 3 Nanjing Institute of Environmental Sciences, Ministry of Ecology and Environment, Nanjing 210042, China

**Keywords:** porous aromatic frameworks, adsorptive desulfurization, π-π interactions, mesopore effect, thiophenic sulfides

## Abstract

The elimination of thiophenic sulfides from fuel oils is essential for both environmental protection and industrial catalysis. However, conventional hydrodesulfurization encounters difficulties due to severe operating conditions and limited efficacy against aromatic heterocyclic sulfur compounds. Adsorptive desulfurization offers notable advantages under milder conditions. In this investigation, topology-guided pore engineering was utilized to fabricate porous aromatic frameworks (PAFs) with distinct pore structures through Suzuki–Miyaura cross-coupling. Notably, PBPAF-2, despite its lower specific surface, demonstrates significantly improved mass transfer kinetics attributed to its unique mesoporous channel (2.13 nm), resulting in notably prolonged dynamic breakthrough retention times compared to other materials in the series. Analysis using synchrotron-assisted FT-IR spectroscopy reveals a blue-shift in benzene ring characteristic peaks following adsorption of dibenzothiophene and benzothiophene, indicating that π-π interactions between electron-rich aromatic rings in PAFs and thiophenic rings are the primary driving force for adsorption. This work proposes a dual-factor synergistic design strategy of “mass transfer optimization–electron cloud matching”, offering a new strategy for the development of highly efficient adsorbents.

## 1. Introduction

The presence of sulfur-containing compounds in fuel oils poses significant challenges to both industrial processes and environmental sustainability. These compounds not only induce the deactivation of industrial catalysts through poisoning and fouling mechanisms but also lead to severe environmental and health issues upon combustion [1,2,3,4,5,6,7]. The release of sulfur oxides (SO_X_) contributes substantially to atmospheric pollution, manifesting as haze formation, acid rain aggravation, and an increased risk of respiratory diseases in exposed populations. Consequently, deep desulfurization has become an imperative processing step before fuel oils can be safely utilized as transportation fuels or chemical feedstocks to meet increasingly stringent environmental regulations worldwide. Currently, hydrodesulfurization (HDS) represents the most widely implemented technology in refineries for sulfur removal. While this catalytic process demonstrates high efficiency in eliminating aliphatic sulfides such as mercaptans and sulfides, it exhibits notably poor efficiency toward refractory aromatic thiophenic compounds, particularly benzothiophene (BT), dibenzothiophene (DBT), and their alkylated derivatives [8,9,10,11]. This technological limitation primarily stems from the exceptional stability of their aromatic heterocyclic structures combined with steric hindrance effects, especially those imposed by methyl groups adjacent to sulfur atoms in compounds like 4,6-dimethyldibenzothiophene. Furthermore, the HDS process necessitates operation under severe conditions, typically requiring temperatures exceeding 300 °C and hydrogen pressures above 4 MPa to achieve satisfactory conversion rates for these stubborn compounds. Such demanding parameters inevitably result in substantial energy consumption, elevated operational costs, and increased safety concerns [12,13,14,15,16].

In response to these challenges, adsorptive desulfurization has emerged as a highly promising alternative technology [17,18,19,20,21,22]. This approach operates under mild conditions, typically at ambient temperature and atmospheric pressure, thereby significantly reducing energy requirements and operational complexity. The fundamental principle relies on selective interactions between adsorbent materials and sulfur-containing molecules through various mechanisms, including π-complexation, acid-base interactions, and direct sulfur-adsorbent coordination. The effectiveness of this technology largely depends on the development of advanced adsorbents with tailored pore structures and surface functionalities.

Porous organic frameworks (POFs) represent a rapidly developing class of porous materials constructed from organic building blocks connected through robust covalent bonds. This family encompasses diverse architectures, including covalent organic frameworks (COFs), conjugated microporous polymers (CMPs), hypercrosslinked polymers (HCPs), and porous aromatic frameworks (PAFs), among others [23,24]. These materials exhibit exceptional characteristics such as tunable pore sizes, high specific surface areas, and remarkable stability—including thermal stability exceeding 400 °C and notable resistance to acidic and basic environments—making them particularly suitable for applications in gas storage, separation processes, purification technologies, and catalytic transformations [25]. Compared to metal–organic frameworks (MOFs), most POFs demonstrate superior chemical stability due to their strong covalent bonding networks, maintaining structural integrity under harsh conditions while allowing extensive functionalization through post-synthetic modification strategies.

Among various POFs, PAFs have attracted considerable attention due to their unique structural features [26,27,28,29]. These materials can be readily synthesized through covalent coupling reactions, constructing multi-aryl building blocks connected by C-C bonds to form diamond-like topologies with inherent porosity. The synthesis flexibility allows for precise control over material properties through careful selection of monomeric units or implementation of post-synthetic modification protocols. For instance, Jing et al. [30] demonstrated that introducing functional groups with high affinity for CO_2_ (e.g., -NH_2_ and -OH) onto tetraphenylmethane-based building blocks significantly enhanced CO_2_ uptake capacity, highlighting the potential for targeted functionalization.

The unique high-density aromatic frameworks of PAFs provide particular advantages for aromatic compound removal. Previous studies have demonstrated their effectiveness in adsorbing various aromatic molecules, including benzene, methanol, and toluene. Building upon these findings, this study proposes an innovative strategy leveraging the electron-rich aromatic skeletons of PAFs to selectively capture thiophenic sulfides through enhanced π-π interactions. We designed and synthesized three novel PAF-type materials via Suzuki–Miyaura cross-coupling reactions using tetra(4-bromophenyl)methane with three different boronic acid monomers: 1,4-phenylenediboronic acid, 4,4′-biphenyldiboronic acid, and 1,1′:4′,1″-terphenyl-4,4″-diboronic acid, designated as PBPAF-1, PBPAF-2, and PBPAF-3, respectively. The structural properties of these synthesized materials were systematically characterized using comprehensive analytical techniques. Their performance in BT and DBT removal was evaluated through both static adsorption measurements and dynamic fixed-bed breakthrough experiments. Furthermore, the adsorption mechanism was elucidated at the molecular level using advanced synchrotron radiation FT-IR spectroscopy, providing fundamental insights into the interaction mechanisms between PAFs and thiophenic compounds. This systematic investigation aims to establish structure-property relationships that can guide the rational design of next-generation adsorbents for deep desulfurization applications, potentially overcoming the limitations of conventional HDS technology while operating under economically favorable conditions.

## 2. Experimental Section

### 2.1. Synthesis of Materials

**Scheme 1:**
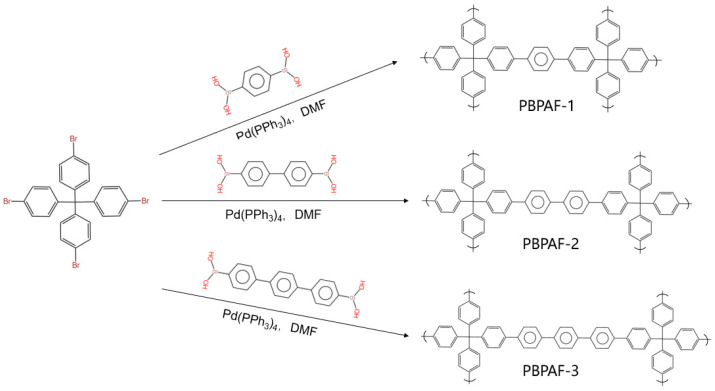
The synthetic process of PBPAF-1, PBPAF-2, and PBPAF-3.

As shown in Scheme 1, the synthesis of PBPAF-1 was carried out through a Suzuki–Miyaura cross-coupling reaction, which represents a robust method for constructing carbon-carbon bonds in porous aromatic frameworks. Specifically, tetrakis(4-bromophenyl)methane (248.04 mg, 0.39 mmol) and 1,4-phenylenediboronic acid (129.28 mg, 0.78 mmol) were precisely weighed and dispersed in 12 mL of anhydrous N,N-dimethylformamide (DMF) within a two-necked flask equipped with a magnetic stirrer. The molar ratio of diboronic acid to tetrabromide was maintained at 2:1 to ensure complete reaction of the bromine functional groups. The reaction system was subjected to nitrogen purging for 30 min to create an oxygen-free environment, which is crucial for preventing oxidation of the boronic acid reagents and ensuring high reaction efficiency. Subsequently, tetrakis(triphenylphosphine)palladium (50 mg, 43 μmol) was added as the catalyst, followed by the introduction of an aqueous solution of K_2_CO_3_ (1.5 mL, 2 M) which served as a base to promote the coupling reaction. The reaction mixture was heated to 150 °C and maintained at this temperature for 24 h under continuous nitrogen protection with constant stirring.

Upon completion of the reaction, the crude product was collected through vacuum filtration and subjected to an extensive washing procedure to remove catalyst residues and inorganic salts. This included sequential washing with tetrahydrofuran (THF), chloroform, and copious amounts of deionized water. To achieve further purification, the product underwent Soxhlet extraction with THF for 24 h, effectively removing any oligomeric species or unreacted starting materials. The final step involved drying under vacuum at 60 °C for 12 h, yielding PBPAF-1 as a dark gray powder with excellent uniformity. For the synthesis of PBPAF-2 and PBPAF-3, identical synthetic protocols were employed with appropriate monomer substitutions. Specifically, 4,4′-biphenyldiboronic acid (188.93 mg, 0.78 mmol) and 1,1′:4′,1″-terphenyl-4,4″-diboronic acid (247.99 mg, 0.78 mmol) were used as coupling partners with tetrakis(4-bromophenyl)methane, respectively.

### 2.2. Materials Characterization

A multi-technique characterization approach was employed to elucidate the structural, morphological, and textural properties of the synthesized PAF materials. Powder X-ray diffraction (XRD) analysis was conducted using a Bruker D8 Advance diffractometer (Bruker, Billerica, MA, USA) equipped with a Cu Kα radiation source (λ = 1.5418 Å) and a nickel monochromator (0.6 mm). The diffraction patterns were systematically recorded in the 2θ range of 5° to 80° with a scanning rate of 10° min^−1^ under operational parameters of 40 kV and 40 mA. This analysis provided critical information regarding the crystallinity and long-range structural ordering of the framework materials.

Fourier transform infrared (FT-IR) spectroscopic measurements were performed on a NICOLET NEXUS-670 spectrometer (Thermofisher Scientific, Waltham, MA, USA) employing the standard KBr pellet method. Prior to analysis, both the sample and KBr matrix were meticulously dried under an infrared lamp for 2 h to eliminate moisture interference. The samples were homogenously mixed with spectroscopic-grade KBr at an optimal mass ratio of 1:100, carefully ground using an agate mortar and pestle to achieve uniform particle size distribution, and compressed into transparent pellets under hydraulic pressure of 10 tons. Spectral acquisition covered the fingerprint region of 640–1000 cm^−1^ with a resolution of 8 cm^−1^, employing 32 accumulated scans to ensure an adequate signal-to-noise ratio for precise peak identification.

Morphological characterization was carried out using field-emission scanning electron microscopy (SEM) on a Hitachi Regulus8100 microscope (Hitachi, Tokyo, Japan) operated at an accelerating voltage of 5 kV and beam current of 10 μA. Sample preparation involved dispersing approximately 1 mg of powder material in 10 mL of anhydrous ethanol through ultrasonication for 15 min, followed by droplet deposition on pristine silicon wafers and natural evaporation under ambient conditions. High-resolution transmission electron microscopy (TEM) was performed on a Thermo Fisher Scientific Talos F200X instrument (Thermo Scientific, Waltham, MA, USA) operating at 200 kV, with sample preparation following an analogous protocol to SEM but utilizing carbon-coated copper grids as substrates.

Textural properties were quantitatively evaluated through N_2_ physisorption measurements at 77 K using a Micromeritics ASAP 2020 analyzer (Micromeritics Instrument Corporation, Norcross, GA, USA). Prior to analysis, approximately 100 mg of each sample underwent degassing under high vacuum conditions (<10^−4^ Pa) at 120 °C for 4 h to ensure complete removal of physisorbed contaminants. The specific surface area was calculated from the adsorption data using the Brunauer–Emmett–Teller (BET) method within the relative pressure range of 0.05–0.30. Total pore volume was determined from the amount of N_2_ adsorbed at P/P_0_ = 0.99, while pore size distribution profiles were derived through nonlocal density functional theory (NLDFT) analysis of the adsorption branch assuming a slit-pore model.

Thermal stability assessment was conducted via thermogravimetric analysis on a NETZSCH STA 449 F5 instrument (NETZSCH-Gerätebau GmbH, Selb, Germany). Approximately 5 mg of sample was placed in an alumina crucible and subjected to programmed heating from 30 to 800 °C at a constant rate of 20 °C min^−1^ under a nitrogen atmosphere with a controlled flow rate of 20 mL min^−1^. Optical properties were investigated using UV-visible spectroscopy on a PerkinElmer Lambda 35 spectrophotometer (PerkinElmer, Waltham, MA, USA), with samples dispersed in anhydrous ethanol via ultrasonication for 5 min and spectra recorded from 300 to 800 nm with 2 nm spectral resolution.

### 2.3. Adsorption Measurements

The desulfurization performance of the PAF materials was systematically evaluated using model fuels prepared with precise concentrations of refractory sulfur compounds. Two separate stock solutions were prepared by weighing designated quantities of DBT and BT into individual 100 mL volumetric flasks, respectively. Each flask was diluted to the mark with isooctane as the solvent and subjected to ultrasonication for 1 h to ensure complete dissolution. The DBT solution was prepared with concentrations ranging from 0 to 750 ppm, while the BT solution was prepared at a fixed concentration of 550 ppm. All solutions were prepared gravimetrically with analytical precision to ensure concentration accuracy.

Adsorption experiments were conducted by introducing 50 mg of accurately weighed adsorbent into 20 mL of model fuel contained in sealed glass vials. The mixtures were agitated continuously for 1 h at room temperature (25 °C) using an orbital shaker operating at 150 rpm to ensure attainment of adsorption equilibrium. Following the adsorption period, the solutions were centrifuged at 10,000 rpm for 10 min to achieve complete phase separation. The sulfur concentration in the supernatant was quantitatively analyzed using an Agilent 7890A gas chromatograph equipped with a flame photometric detector (FID) specifically optimized for sulfur detection. The GC separation was achieved using an HP-5 capillary column (30 m length, 0.32 mm diameter, 0.25 μm film thickness) with high-purity helium as carrier gas at a flow rate of 1.0 mL min^−1^.

Competitive adsorption studies were conducted by adding 15 vol% toluene to the model fuel containing 550 ppm of both DBT and BT during solution preparation, resulting in a mixed model fuel system. A measured amount of adsorbent, previously activated at 150 °C, was dispersed in 10 mL of the mixed model fuel. The mixture was ultrasonicated to achieve complete dispersion and then allowed to stand for 1 h. The supernatant was subsequently collected for quantitative analysis using gas chromatography.

Dynamic adsorption characteristics were evaluated using a fixed-bed adsorption system comprising a precision syringe pump, a temperature-controlled adsorption column, and automated fraction collectors. Specifically, 150 mg of adsorbent was activated at 150 °C for 10 h under nitrogen flow to remove physisorbed species, then packed into a quartz reactor (4 mm internal diameter × 200 mm length) with quartz sand layers at both ends to ensure uniform flow distribution. The model fuel containing 550 ppm each of DBT and BT in isooctane was delivered upward through the adsorption bed at controlled flow rates using a syringe pump, with effluent samples collected at predetermined time intervals for subsequent GC analysis. The breakthrough experiments continued until complete saturation was achieved, as indicated by constant sulfur concentration in the effluent stream. Desorption behavior was subsequently investigated by switching the feed to pure isooctane (50 mL) under identical hydrodynamic conditions.

## 3. Results and Discussion

### 3.1. Structural Characterization

**Figure 1:**
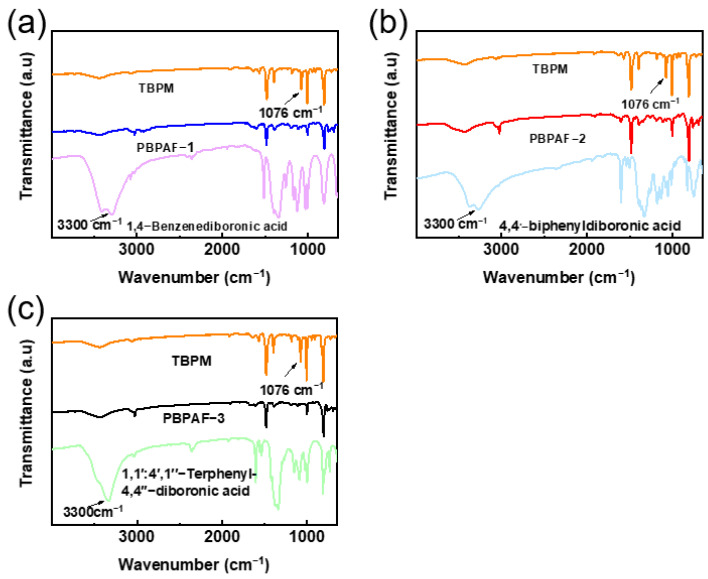
FT-IR spectra of (**a**) PBPAF−1, (**b**) PBPAF−2, and (**c**) PBPAF−3 (include the reactants).

FT-IR spectroscopy was systematically employed to elucidate the chemical bonding evolution and confirm the successful construction of porous frameworks via the Suzuki–Miyaura coupling reaction. The spectral comparison between precursors and resulting polymers provides compelling evidence for molecular-level structural transformations. As illustrated in Figure 1, the precursor tetrabromotetraphenylmethane (TBPM) displays a distinct absorption band at 1076 cm^−1^, characteristic of C-Br stretching vibrations, while the three diboronic acid monomers (1,4-phenylenediboronic acid, 4,4′-biphenyldiboronic acid, and 1,1′:4′,1″-terphenyl-4,4″-diboronic acid) exhibit broad vibrational bands centered around 3300 cm^−1^, corresponding to B-OH functional groups. The complete disappearance of these characteristic peaks in the spectra of PBPAF-1, PBPAF-2, and PBPAF-3 unequivocally confirms the efficient consumption of both bromo and boronic acid functionalities during the polycondensation process. This spectroscopic evidence demonstrates nearly quantitative conversion efficiency in the covalent bond formation, leading to fully cross-linked polymeric networks without detectable residual monomers or incomplete reactions. The successful elimination of these precursor signatures establishes a fundamental foundation for subsequent structural analyses and confirms the molecular-level integrity of the synthesized frameworks.

**Figure 2:**
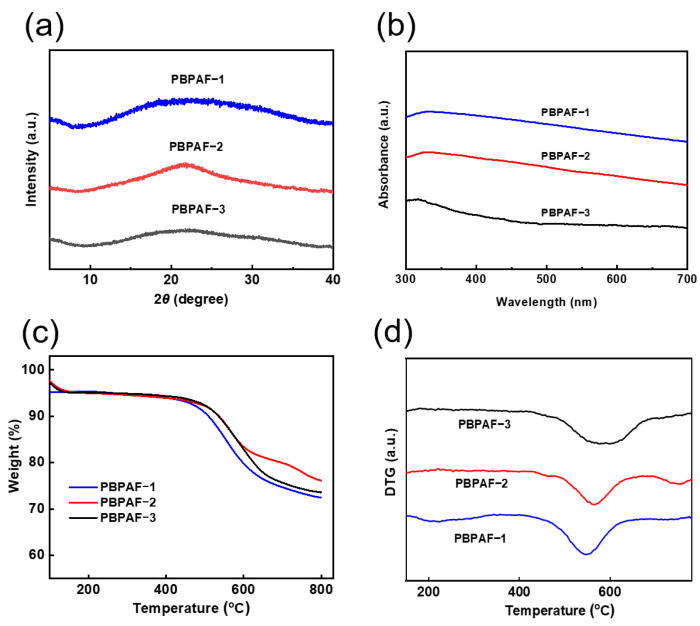
(**a**) Wide−angle XRD, (**b**) UV−vis spectra, (**c**) TG, and (**d**) DTG curves of PBPAF−1, PBPAF−2, and PBPAF−3.

The structural order and crystallinity of the synthesized materials were comprehensively investigated through X-ray diffraction analysis. As revealed in Figure 2a, all materials exhibit broad diffraction halos within the 2*θ* range of 5–40°, devoid of any sharp Bragg diffraction peaks. This pattern indicates amorphous polymeric structures, which aligns with the typical characteristics of hypercrosslinked porous aromatic frameworks. The absence of crystalline domains suggests the formation of isotropic network structures with random orientation of building blocks, resulting from the three-dimensional nature of the tetrahedral tetraphenylmethane nodes connected through rigid aromatic linkers. To further characterize the electronic properties, complementary UV-visible absorption spectra (Figure 2b) reveal weak but distinct absorption bands between 300 and 400 nm for all samples. This can be attributed to the conjugated nature arising from the high-density benzene ring structures in these three PAFs [31]. Moreover, the similar absorption profiles among the three materials suggest comparable electronic structures despite variations in linker length. To assess another key material property, the thermal stability of the PAF materials was systematically investigated through thermogravimetric analysis under a nitrogen atmosphere. As presented in Figure 2c, all three polymers exhibit similar decomposition patterns with three distinct weight loss stages. The initial minor mass loss below 150 °C (approximately 3–5%) corresponds to the removal of physically adsorbed water, residual solvent molecules trapped within the pores, and moisture absorbed during sample handling. Notably, the exceptional thermal stability of these aromatic frameworks is demonstrated by the major decomposition step occurring between 400 and 600 °C, with DTG peaks observed at 523 °C, 538 °C, and 551 °C for PBPAF-1, PBPAF-2, and PBPAF-3, respectively (Figure 2d). This high-temperature decomposition corresponds to the breakdown of covalent C-C bonds in the aromatic frameworks and subsequent carbonization processes. Furthermore, the progressive increase in decomposition temperature from PBPAF-1 to PBPAF-3 correlates with the extending conjugation length in the molecular building blocks, with PBPAF-3 exhibiting the highest thermal stability due to the terphenyl units providing enhanced π-conjugation and molecular rigidity. Overall, the outstanding thermal stability, particularly the maintenance of structural integrity below 300 °C, ensures that these materials can withstand the temperature conditions encountered in various industrial applications, including adsorptive desulfurization processes where thermal regeneration may be required.

**Figure 3:**
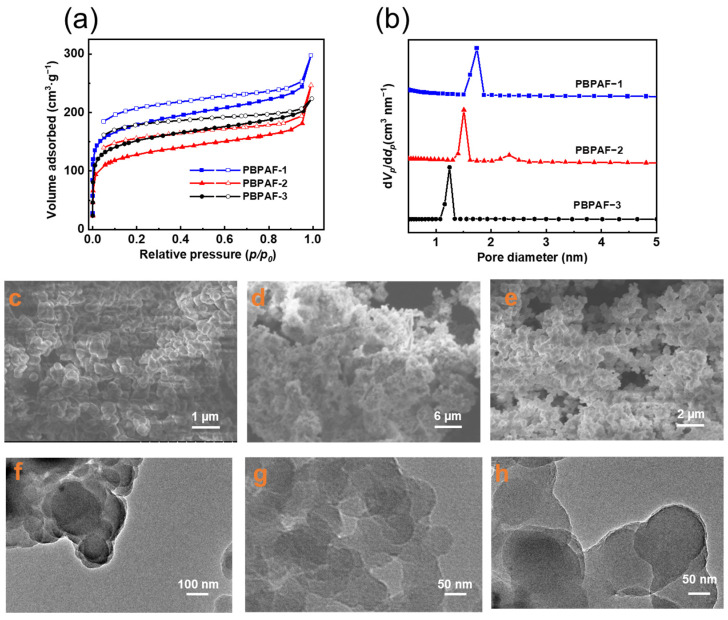
(**a**) N_2_ adsorption and desorption curves and (**b**) pore size distribution of PBPAF−1, PBPAF-2, and PBPAF-3. SEM images of (**c**) PBPAF−1, (**d**) PBPAF−2, and (**e**) PBPAF−3. TEM images of (**f**) PBPAF−1, (**g**) PBPAF−2, and (**h**) PBPAF−3.

The porous properties were quantitatively evaluated through N_2_ physisorption measurements at 77 K. As shown in Figure 3a, all three materials exhibit Type I adsorption isotherms according to the IUPAC classification, characterized by rapid nitrogen uptake at low relative pressures (P/P_0_ < 0.01) followed by nearly horizontal plateaus at higher pressures. This behavior is typical of microporous materials. The sharp uptake at low pressures indicates the presence of narrow micropores with strong gas–solid interactions, while the limited hysteresis between adsorption and desorption branches suggests minimal mesoporosity. The corresponding pore size distributions derived from NLDFT calculations (Figure 3b) confirm the microporous nature, with PBPAF-1 showing a primary pore size maximum at 1.7 nm, while PBPAF-3 exhibits a narrower distribution centered at 1.24 nm. Interestingly, PBPAF-2 demonstrates a bimodal distribution with micropores at 1.3 nm and mesopores centered at 2.13 nm, indicating structural heterogeneity introduced by the biphenyl linker. It should be noted that the pore size distribution derived from NLDFT calculations using the slit-pore model provides an estimation, as this model represents a simplified approximation of the actual pore topology in these amorphous frameworks. The structural heterogeneity observed in PBPAF-2 originates from the rotational flexibility of the biphenyl linker around its central C–C bond. This inherent conformational freedom results in a wider distribution of dihedral angles during the cross-linking process, leading to a heterogeneous network architecture. Within this framework, regions of efficient molecular packing give rise to micropores, while zones of less dense packing create mesoporous voids, as evidenced by the distinct 2.13 nm mesopores identified in the pore size distribution. In contrast, the terphenyl linker employed in PBPAF-3 exhibits significantly greater rigidity and linearity due to its extended π-conjugated system. This molecular constraint suppresses conformational diversity and promotes a more regular and densely packed framework, ultimately favoring the formation of a predominantly microporous structure with uniform pore dimensions. The textural parameters summarized in Table 1 reveal that PBPAF-1 possesses the highest specific surface area (616 m^2^·g^−1^) and pore volume (0.63 cm^3^·g^−1^), followed by PBPAF-3 (554 m^2^·g^−1^, 0.59 cm^3^·g^−1^). Detailed analysis of pore volumes confirms that PBPAF-2 possesses a significant mesopore volume of 0.08 cm^3^·g^−1^ alongside a micropore volume of 0.44 cm^3^·g^−1^, substantiating its composite micro-mesoporous character. The progressive decrease in surface area with increasing linker length suggests that longer bridging units may lead to more efficient packing or increased framework flexibility, resulting in reduced accessible porosity.

Beyond the porous properties, the morphological features of the synthesized PAFs were examined using electron microscopy techniques. SEM and TEM images (Figure 3) reveal that all three materials possess well-defined spherical morphologies with narrow size distributions. PBPAF-1 exhibits a well-defined spherical morphology with particle sizes of approximately 200 nm (Figure 3c,f). Similarly, both PBPAF-2 and PBPAF-3 materials are composed of uniform spherical particles, with diameters of about 100 nm and 150 nm, respectively (Figure 3d,e,g,h). The morphological consistency across different samples suggests similar nucleation and growth mechanisms during the polymerization process, while the size variations may be attributed to differences in solubility and reactivity of the respective diboronic acid monomers. The spherical morphology provides favorable characteristics for practical applications, including good flowability and packing density in adsorption columns. The amorphous nature observed in XRD patterns is further corroborated by the absence of lattice fringes in high-resolution TEM images, consistent with the expected disordered network structures.

### 3.2. Desulfurization Performance

The adsorption desulfurization performance of the synthesized PAF materials was systematically evaluated using BT and DBT as representative refractory sulfur compounds. These molecules possess distinct structural characteristics, with BT exhibiting a slightly distorted aromatic system and DBT maintaining a completely planar configuration due to its additional fused benzene ring. Their kinetic diameters were determined to be 6.5 × 8.9 Å for BT and 6.07 × 9.81 Å for DBT, respectively (Table 2). Critical analysis of the pore structural parameters reveals that all three PAFs possess adequate porosity to accommodate these sulfur compounds. The minimum pore sizes (1.7 nm for PBPAF-1, 1.24 nm for PBPAF-3) significantly exceed the molecular dimensions of both adsorbates, while the substantial pore volumes (0.59–0.63 cm^3^·g^−1^) provide sufficient space for unhindered diffusion and accommodation of multiple sulfur molecules. More importantly, the planar and rigid molecular structure of DBT enables more efficient π-π stacking interactions with the electron-rich aromatic pore walls of the PAFs compared to the slightly non-planar BT configuration. This fundamental geometric distinction establishes a structural basis for the differential adsorption behaviors observed in subsequent experiments.

**Figure 4:**
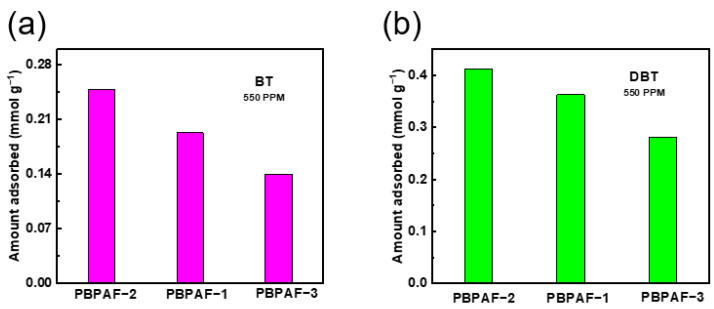
The adsorption performance of PBPAF adsorbents for (**a**) BT and (**b**) DBT.

The equilibrium adsorption capacities were quantitatively determined through static adsorption experiments using model fuels containing 550 ppm of individual sulfur compounds. As comprehensively summarized in Figure 4, the three PAF adsorbents displayed markedly different affinities toward BT and DBT molecules. For BT adsorption, the PBPAF-3 adsorbent exhibited the lowest saturated adsorption capacity of approximately 0.14 mmol·g^−1^. In contrast, the PBPAF-2 adsorbent showed the highest capacity of about 0.24 mmol·g^−1^, which is nearly double that of PBPAF-3. This trend was more pronounced for DBT adsorption. PBPAF-2 exhibited an exceptional capacity of 0.41 mmol·g^−1^, which is 46.4% higher than that of PBPAF-3 (0.28 mmol·g^−1^) and surpasses that of PBPAF-1 (0.35 mmol·g^−1^). The consistently enhanced performance of PBPAF-2 toward both sulfur compounds suggests the presence of favorable structural features beyond mere surface area considerations. When benchmarked against other PAF-type adsorbents reported in the literature (Table 3), the performance of our materials is on par with that of other materials for the removal of thiophene-based sulfides.

**Figure 5:**
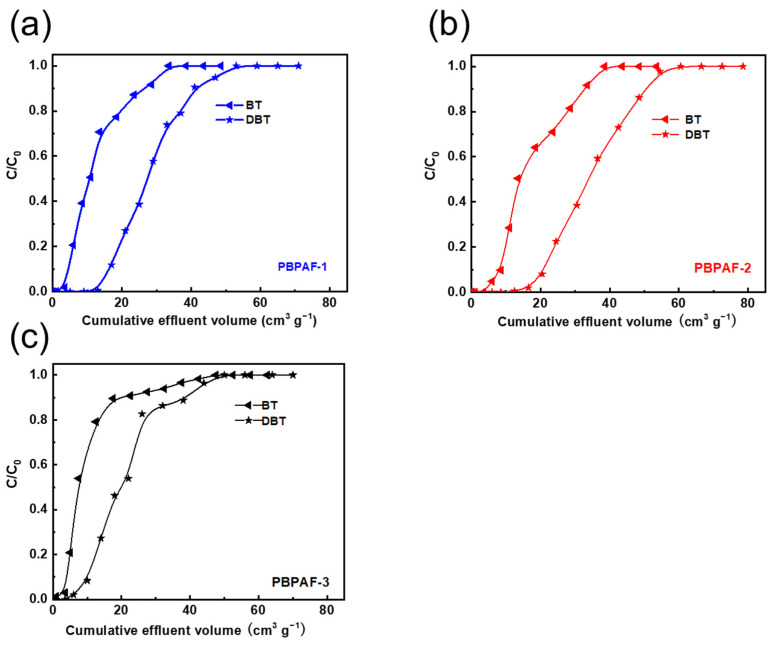
Dynamic breakthrough curves for BT and DBT of (**a**) PBPAF-1, (**b**) PBPAF-2, and (**c**) PBPAF-3.

To assess the practical applicability under dynamic conditions, fixed-bed breakthrough experiments were conducted with continuous flow of model fuel. As illustrated in Figure 5, all three adsorbents exhibited substantially longer breakthrough times for DBT compared to BT, confirming the stronger interaction between DBT molecules and the aromatic frameworks. Additionally, the saturated adsorption capacities of the three materials were determined by the integration method, with the results summarized in Table 3, which are consistent with the static adsorption experiments. The PBPAF-1 adsorbent exhibited higher saturated adsorption capacities for both DBT and BT molecules compared to the PBPAF-3 adsorbent. This superior performance is likely attributable to the larger specific surface area of PBPAF-1, which provides a greater contact area and thereby enhances interactions with the sulfide molecules. While PBPAF-1 demonstrated expectedly higher capacities than PBPAF-3 due to its larger surface area, the exceptional performance of PBPAF-2 deserves particular attention. Despite possessing the lowest specific surface area (463 m^2^·g^−1^), PBPAF-2 outperformed PBPAF-1 by 17.1% for DBT and 33.3% for BT adsorption. This apparent anomaly can be rationally explained by its unique hierarchical pore structure containing 2.13 nm mesopores, which facilitate superior mass transfer kinetics and improve accessibility to adsorption sites. The reduced diffusion resistance allows for more efficient utilization of the available surface area, highlighting the critical importance of mesopore engineering in optimizing adsorbent performance.

Further analysis of the structure-performance relationship reveals that the enhanced adsorption capacity of PBPAF-2 originates from the synergistic combination of adequate micropores for strong π-π interactions and mesopores for efficient molecular transport. The biphenyl linker in PBPAF-2 appears to create an optimal balance between porosity accessibility and adsorption energy, whereas the shorter phenyl linker in PBPAF-1 and longer terphenyl linker in PBPAF-3 lead to less ideal pore architectures for thiophenic compounds. This understanding provides valuable guidance for the molecular design of advanced adsorbents targeting specific sulfur compounds in fuel desulfurization applications. Preliminary desorption studies further support the adsorption mechanism, demonstrating that over 90% of adsorbed DBT and BT can be readily desorbed using pure isooctane, confirming the highly reversible nature of the physisorption process. However, it should be noted that the current study primarily establishes the fundamental structure-property relationships and single-cycle performance, while the long-term cyclic stability and regeneration efficiency under multiple adsorption–desorption cycles remain to be systematically investigated in future work. This understanding provides valuable guidance for the molecular design of advanced adsorbents targeting specific sulfur compounds in fuel desulfurization applications, while also highlighting the need for further studies on material regenerability for practical implementation.

### 3.3. Investigation of Adsorption Mechanism

**Figure 6:**
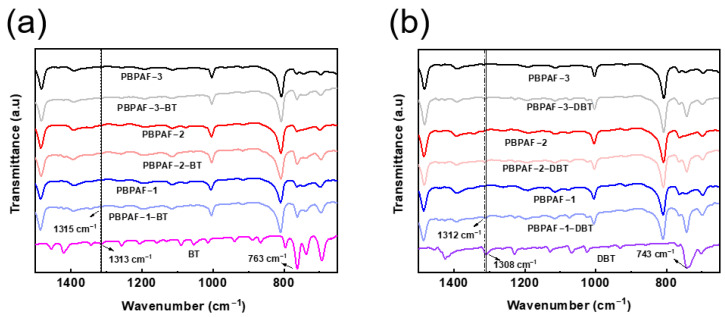
FT-IR spectra of PAF adsorbents for (**a**) BT and (**b**) DBT (before and after adsorption saturation).

To investigate the adsorption mechanisms of BT and DBT molecules on three porous materials (PBPAF-1, PBPAF-2, and PBPAF-3), FT-IR spectroscopy was performed on the adsorbents after reaching saturation, with the results presented in Figure 6. For BT, the peak observed at 763 cm^−1^ corresponds to the S–C stretching vibration, while the peak at 1313 cm^−1^ is attributed to the aromatic ring stretching vibration of the BT molecule. After BT adsorption, the aromatic ring stretching vibration shifted from 1313 cm^−1^ to 1315 cm^−1^, representing a blue shift of approximately 2 cm^−1^ (Figure 6a). In the case of DBT, the peak at 743 cm^−1^ is assigned to the S–C stretching vibration, and the peak at 1308 cm^−1^ corresponds to the aromatic ring stretching vibration. Like BT adsorption, the aromatic ring vibration of DBT shifted from 1308 cm^−1^ to 1312 cm^−1^ after adsorption, showing a larger blue shift of 4 cm^−1^. Notably, no shift was observed in the S–C vibrational band for either molecule (Figure 6b). These results suggest that the adsorption of BT and DBT on these porous materials is primarily mediated by π–π interactions between the aromatic rings in the adsorbents and those in the thiophene-based sulfides. Compared to BT, DBT possesses an additional aromatic ring, leading to a greater number of π-electrons and thus a higher electron cloud density. As a result, the π-electron density of BT is lower than that of DBT, weakening the π–π interactions between the adsorbents and BT molecules. This accounts for the smaller wavenumber shift observed for BT and explains why all three PAFs exhibit higher adsorption capacities for DBT than for BT.

The established mechanism highlights the importance of molecular design strategies that enhance π-electron density and optimize pore geometry for developing advanced adsorbents for deep desulfurization applications. These findings provide fundamental guidance for the rational design of next-generation porous materials targeting specific sulfur compounds through precise control of π-π interactions and molecular recognition capabilities.

## 4. Conclusions

This study successfully synthesized three porous aromatic frameworks (PBPAF-1, PBPAF-2, and PBPAF-3) via Suzuki–Miyaura cross-coupling. Structural analysis confirmed their amorphous microporous nature, high surface areas (463–616 m^2^·g^−1^), uniform spherical morphology (100–200 nm), and excellent thermal stability (>400 °C). A distinct mesopore (2.13 nm) in PBPAF-2 was found to facilitate adsorption. In both static and dynamic tests, PBPAF-2 showed superior capture of DBT (0.41 mmol·g^−1^) and BT (0.24 mmol·g^−1^), exceeding surface area-based predictions. Moreover, the dynamic adsorption breakthrough time of PBPAF-2 surpassed that of PBPAF-3 by a factor of 2.1. Synchrotron FT-IR results indicated that adsorption is governed by π–π interactions, with a larger blue shift for DBT (4 cm^−1^) than BT (2 cm^−1^), suggesting stronger orbital coupling with the more conjugated system. The absence of a shift in the S–C vibrational band further corroborated that the adsorption selectivity is governed by electron cloud compatibility between the aromatic rings. In conclusion, this work proposes a novel dual-factor synergistic strategy integrating “mesopore-mediated mass transfer optimization” and “π-electron density matching,” thereby providing a rational design pathway for developing high-performance adsorbents toward efficient deep desulfurization.

## Figures and Tables

**Table 1:** Physical parameters of synthetic materials.

Sample	S_BET_ (m^2^·g^−1^)	Micropore Volume (cm^3^·g^−1^)	Mesopore Volume (cm^3^·g^−1^)	Pore Size (nm)
PBPAF−1	616	0.63	-	1.7
PBPAF−2	463	0.44	0.08	1.51, 2.13
PBPAF−3	554	0.59	-	1.24

**Table 2:** Structure and properties of the aromatic sulfides used in the experiments.

Sulfide	Chemical Formula	Structure	Molecular Size (Å)
BT	C_8_H_6_S	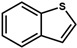	6.5 × 8.9
DBT	C_12_H_8_S	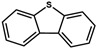	6.07 × 9.81

**Table 3:** Comparison of adsorption desulfurization capacity of different adsorbents.

Adsorbent	Model Oil	T/°C	Adsorbate	Adsorption Capacity/mmol·g^−1^	Ref.
PBPAF−2	Isooctane	25 °C	DBT	0.41	This work
PBPAF−2	Isooctane	25 °C	BT	0.24	This work
PBPAF−1	Isooctane	25 °C	DBT	0.36	This work
PBPAF−1	Isooctane	25 °C	BT	0.19	This work
PBPAF−3	Isooctane	25 °C	DBT	0.28	This work
PBPAF−3	Isooctane	25 °C	BT	0.14	This work
HY/SBA−15	Nonane	25 °C	TH	0.09	[32]
Ce-HY/SBA−15	Nonane	25 °C	TH	0.16	[32]
CTAB−H−0.3	n-octane	20 °C	BT	0.42	[8]
ACO	Petroleum ether	30 °C	DBT	0.22	[33]
AgNO_3_-SBA−15	n-Decane	25 °C	DBT	0.21	[34]
CuCl/AS−10	Isooctane	25 °C	BT	0.25	[35]
Na/Y	Isooctane	25 °C	DBT	0.27	[35]
Cu_2_O/MIL−101(Cr)	Isooctane	25 °C	BT	0.31	[36]

## Data Availability

Data is contained within the article.
